# Duration-dependent acute effects of foam rolling on dominant-hand visual reaction time in male youth basketball players: A randomized controlled study

**DOI:** 10.1371/journal.pone.0358357

**Published:** 2026-09-22

**Authors:** Koray Samet Altınçekiç, Şevval Çelen, Osman Coşkun, Sinan Avcı, Selin Biçer Baikoğlu, Meltem Özağır, Suzan Dal, Orkun Akkoç

**Affiliations:** 1 Department of Coaching Education, Faculty of Sports Sciences, Istanbul Gedik University, Istanbul, Türkiye; 2 Fenerbahçe Sports Club, Basketball Department, Istanbul, Türkiye; 3 Department of Anatomy, Istanbul Faculty of Medicine, Istanbul University, Istanbul, Türkiye; 4 Department of Sports Heath Sciences, Faculty of Sports Sciences, Istanbul University-Cerrahpasa, Istanbul, Türkiye; 5 Department of Recreation, Faculty of Sports Sciences, Istanbul Gelisim University, Istanbul, Türkiye; 6 Department of Exercise and Sport Sciences on Disabilities, Faculty of Sports Sciences, Istanbul University-Cerrahpasa, Istanbul, Türkiye; 7 Department of Movement and Training Sciences, Faculty of Sports Sciences, Istanbul University-Cerrahpasa, Istanbul, Türkiye; Instituto Politecnico de Viana do Castelo, PORTUGAL

## Abstract

**Objective:**

This study examined the acute effects of different foam rolling (FR) durations on dominant-hand visual reaction time in male youth basketball players.

**Methods:**

In this parallel-group randomized trial, 102 U16–U18 players were allocated equally to FR-A (30 seconds per region), FR-B (60 seconds per region), or passive rest. Five valid trials were recorded before and after the intervention. The lowest value was the primary best-trial score; the mean and median were sensitivity measures. Baseline-adjusted post-test differences were assessed using ANCOVA. Because the standard best-trial ANCOVA violated slope-homogeneity and linearity assumptions, an extended nonlinear baseline-interaction model was evaluated using 5,000 bias-corrected and accelerated bootstrap replicates. Mixed-design ANOVAs examined pre-to-post trajectories.

**Results:**

FR-A had lower adjusted post-test reaction time than FR-B under best-trial (difference = −21.86 ms, 95% CI: −30.31 to −13.41; adjusted p < .01), mean-based (−12.36 ms, adjusted p < .01), and median-based scoring (−10.94 ms, adjusted p = .03). The FR-A–control difference was significant in the standard best-trial ANCOVA (−15.44 ms, 95% CI: −24.12 to −6.76; adjusted p < .01) and extended bootstrap comparison (−14.79 ms, adjusted p < .01), but not in mean-based (adjusted p = .33) or median-based analyses (adjusted p = .39). Extended bootstrap estimates were also higher in FR-B than in the control group (9.52 ms, adjusted p = .01). Because this model contained baseline interactions, these contrasts represented average rather than constant differences across baseline values. The time × group interaction was significant only for best-trial scoring (p < .01).

**Conclusion:**

FR-A had consistently lower adjusted reaction time than FR-B across all scoring methods. However, the FR-A–control comparison depended on baseline reaction time and scoring method. Thus, the findings do not establish a scoring-independent benefit of the 30-second protocol over passive rest or support a firm practical recommendation.

## Introduction

Fascial tissues are mechanically responsive and richly innervated connective structures that may contribute to acute neuromuscular outcomes through proprioceptive feedback, sensory modulation, and force transmission. [1–3] It has been suggested that modulation of fascial tissues through mechanical stimulation may influence tissue viscoelasticity, local circulation, and sensory feedback, which may in turn exert indirect effects on neuromuscular performance [4,5]. Understanding these tissue-level responses within a broader applied-biomechanics framework may contribute to the optimization of athletic performance and inform strategies aimed at injury-risk mitigation and athlete rehabilitation [6]. Since fascia is densely innervated by mechanoreceptors that are sensitive to manipulation, increased afferent input may reduce sympathetic activation in the central nervous system and consequently decrease myofascial tone [4,5,7–9]. In contemporary sport science literature, it has become increasingly clear that performance cannot be explained solely by traditional parameters such as muscular strength and cardiovascular endurance [10–13]. In high-tempo team sports such as basketball, performance depends not only on well-developed physical capacities but also on the effectiveness of perceptual and cognitive processes [14,15]. In fast-paced team sports such as basketball, reaction time to visual stimuli is relevant to athletic performance [16,17]. Accordingly, visual reaction time can be used as a practical laboratory-based measure of response speed under standardized conditions. In fast-paced and unpredictable sports such as basketball, the ability of athletes to respond rapidly and accurately to environmental stimuli is critically important for performance [18]. At this point, self-myofascial release (SMR), particularly when performed using a foam roller (FR), emerges as a fascial stimulation method that is widely preferred by athletes [19]. FR has been shown to produce various acute effects, such as increasing range of motion, reducing muscle stiffness, improving local blood flow, and modulating pain perception [20,21]. However, despite the potential influence of the fascial system on neuromuscular performance, studies examining the effects of FR on psychomotor performance outcomes, particularly reaction time, remain limited. In the existing literature, studies examining the effects of fascial stimulation on reaction-time-related outcomes have mostly focused on therapist-applied interventions, such as manual myofascial release or fascial manipulation [22,23]. However, foam rolling is a self-administered form of self-myofascial release that can be performed independently by athletes and may therefore be more feasible for field-based sport settings. Despite this practical relevance, the acute effects of foam rolling on visual reaction time in athletes remain insufficiently investigated. To contextualize the duration-specific design of the present study, a focused narrative search was conducted during manuscript preparation in PubMed and Google Scholar using combinations of the terms “foam rolling,” “self-myofascial release,” “duration,” “reaction time,” “acute performance,” and “neuromuscular performance.” No publication-date restriction was applied. English-language, peer-reviewed publications were considered eligible when they addressed at least one of the following topics: foam rolling or related myofascial interventions, intervention duration, reaction time, or acute performance and neuromuscular outcomes. Non-English and non-peer-reviewed publications and sources without direct relevance to these topics were not considered. Because this search was intended to provide focused narrative context rather than a systematic evidence synthesis, no formal restrictions were applied according to study design, population, acute versus chronic intervention, or therapist- versus self-administered techniques; no protocol registration, exhaustive screening procedure, or formal risk-of-bias assessment was undertaken. Within this literature, systematic reviews and human primary studies reporting SMR/FR application duration and acute performance-related outcomes were prioritized when developing the duration-specific rationale. Previous studies have used heterogeneous SMR/FR application durations, commonly ranging from 30 seconds to 1 minute, with examples including [24–27]. This variability in treatment parameters makes it difficult to determine whether shorter or longer FR applications produce more favorable acute responses. Therefore, comparing 30-second and 60-second FR protocols may help clarify whether the acute response of visual reaction time differs according to application duration. Kuruma et al. reported that MFR applied to the quadriceps and hamstring muscles produced positive effects on premotor time and total reaction time [22], while Sawamura and Mikami reported that fascial manipulation applied to the right brachial fascia improved neuromuscular response parameters such as motor time and total reaction time [23]. However, the acute effects of self-administered FR protocols targeting whole-body fascial lines on reaction time in athletes have not yet been sufficiently clarified. This gap makes it necessary to examine the effects of duration-specific FR applications on reaction time. Based on this duration-dependent rationale, it was hypothesized that the 30-second FR protocol would produce a more favorable acute response in visual reaction time than the control condition, whereas the 60-second FR protocol might not provide additional benefit and could be associated with less favorable responses. This assumption was based on the possibility that shorter applications may provide sufficient sensory stimulation, while longer applications may induce greater inhibitory or fatigue-like responses. Therefore, the planned primary comparisons were FR-A versus control and FR-A versus FR-B. Accordingly, the aim of this study was to examine the duration-dependent acute effects of FR on dominant-hand visual reaction time in male youth basketball players.

## Materials and methods

### Study design

This study was designed as a parallel-group, randomized controlled experimental study to examine the acute effects of different durations of foam rolling (FR) on visual reaction time in male youth basketball players. The study included two intervention groups and one control group: the FR-A group, which performed FR for 30 seconds per region; the FR-B group, which performed FR for 60 seconds per region; and the control group, which performed passive rest.

The primary estimand was defined as the between-group difference in dominant-hand post-test best-trial reaction time adjusted for the corresponding pre-test best-trial value. The best-trial score was defined as the lowest reaction time obtained across the five valid trials. To evaluate whether the findings were sensitive to this scoring approach, the arithmetic mean and median of the same five valid trials were also analyzed separately for the pre-test and post-test measurements. Mixed-design analyses of the raw pre-to-post trajectories were conducted as complementary analyses and were not used to replace the primary baseline-adjusted estimand. Pre-test to post-test change scores were calculated and presented descriptively to indicate the direction and magnitude of the observed changes.

The study protocol was conducted in two stages. In the first stage, all participants completed a familiarization session covering the testing environment and foam roller procedures. In the second stage, the experimental testing session was conducted two weeks after the familiarization session. During the experimental session, participants were tested individually. Each participant first completed the pre-test visual reaction time assessment, followed by the intervention or control protocol according to group allocation, and then the post-test assessment. Participants in both FR groups began the post-test after a standardized 60-second rest period following completion of their respective FR protocols to assess the acute response to each protocol. The FR-A group was not given additional passive rest to match the total duration of FR-B because such a delay would have introduced a recovery interval between completion of the 30-second protocol and post-test assessment, thereby altering the intended immediate post-intervention measurement window. The control group completed seated passive rest equivalent to the total application time of FR-B, which represented the longer intervention condition. Consequently, the elapsed time between the pre-test and post-test assessments was matched between FR-B and the control group but was shorter in FR-A. Exact participant-level pre-test-to-post-test intervals were not recorded.

### Participants

A total of 102 male youth basketball players competing in the U16 and U18 age categories voluntarily participated in the study. The participants consisted of athletically active individuals who regularly attended team training sessions 3–4 days per week and had maintained regular physical activity during the previous six months. Participants were asked to continue their usual training activities throughout the study period. Participants were equally allocated into three groups: the FR-A group, the FR-B group, and the control group. Participants were randomly allocated to the FR-A, FR-B, or control group in a 1:1:1 ratio using block randomization with block sizes of 6 and 9. The allocation sequence was generated by an external statistician who was not otherwise involved in participant recruitment, eligibility assessment, intervention delivery, data collection, or statistical analysis. Group assignments were placed in sequentially numbered, opaque, sealed envelopes. The principal investigator assessed participant eligibility according to the predefined inclusion and exclusion criteria and enrolled eligible participants in the study. After eligibility had been confirmed and the participant had been enrolled, the next envelope in numerical order was opened, and the participant was assigned to the group indicated inside the envelope. The principal investigator did not have access to the allocation sequence before participant enrolment and therefore could not know the upcoming group assignment. Because of the nature of the intervention, participants and the investigator administering the protocols could not be blinded to group allocation. However, group identities were coded during data entry and statistical analysis. Each group included 34 athletes. Participants’ ages ranged from 15 to 18 years, with a mean age of 15.95 ± 0.88 years, a mean height of 182.06 ± 8.08 cm, and a mean body mass of 74.84 ± 10.98 kg. The inclusion criteria were as follows: being a licensed basketball player with a valid license for the 2024–2025 competitive season, having at least two years of regular training experience, having no pathology related to the cardiovascular or pulmonary system, having no acute or chronic sports injury that could prevent participation in the study, being a non-smoker, and maintaining normal dietary habits on the testing day. In addition, participants were asked not to use analgesics or pharmacological agents that could affect performance during the study period. The exclusion criteria were defined as the use of performance-enhancing doping substances or ergogenic aids, chronic sports injury or orthopedic pathology, alcohol consumption within the previous 24 hours, consumption of caffeine or stimulant-containing products within the previous 2 hours, high-intensity physical activity within the 48 hours before the testing sessions, insufficient sleep duration, reported excessive fatigue, and the use of therapeutic medication or analgesics. A health history questionnaire was administered to identify medical conditions that could affect participation in the study. Recent injuries involving muscle, bone, tendon, ligament, or connective tissue were taken into consideration. Participant recruitment and data collection were conducted between 2 May 2025 and 13 June 2025. Before participation, all participants were provided with detailed information regarding the study procedures, potential benefits, and possible risks. Written informed consent was obtained directly from participants who were 18 years old. For participants under 18 years of age, written informed consent was obtained from their parents or legal guardians prior to participation. In addition, participants were provided with written explanations of the foam rolling application protocol and the reaction time testing procedure. The study was approved by the Istanbul University-Cerrahpasa Non-Interventional Clinical Research Ethics Committee, approval number: 2025/187. All procedures were conducted in accordance with the Declaration of Helsinki and relevant national ethical guidelines.

### Procedure

#### Familiarization.

Two weeks before the experimental session, all participants completed a familiarization session covering the testing environment and foam roller procedures. During this session, participants were familiarized with the screen position, mouse placement, general response instructions and foam roller techniques and the key points of the applications were demonstrated. No reaction-time stimuli were presented, and no test trials were completed during this session. This session did not include exposure to or practice with the specific Human Benchmark Reaction Time Test. The specific name and web source of the test were not disclosed to minimize the possibility of unsupervised practice before the experimental session. Supervised practice trials were provided only on the experimental testing day, immediately before the recorded trials. During the familiarization session, demographic and anthropometric data, including age, height, and body mass, were also recorded. Two weeks later, participants attended the experimental testing session. Participants were instructed to consume the same or similar meals during the two days preceding testing to reduce the potential influence of differences in carbohydrate or caffeine intake on performance. All tests were conducted in the same location and supervised by the same researchers.

#### Foam rolling protocol.

A Delta foam roller (model/stock code: FBE48) was used in the study. The foam roller was made of high-density, medium-firmness EVA material, had a textured massage surface, and measured 33 cm in length and 14 cm in diameter. Foam rolling applications were performed to cover whole-body fascial lines. The application sequence included the calf group, hamstring group, upper fibers of the hip complex, hip rotators, iliotibial band, adductor group, quadriceps complex, thorax, latissimus dorsi, and pectoralis major regions, respectively. Except for the thoracic region, the upper fibers of the hip complex, and the m. quadriceps femoris, each region was treated bilaterally. In the FR-A group, foam rolling was applied to each region for 30 seconds at a tempo of 40 BPM, with 30 seconds of rest between sets. The total application time for this group was approximately 10–12 minutes. In the FR-B group, foam rolling was applied to each region for 60 seconds at a tempo of 40 BPM, with 30 seconds of rest between sets, and the total application time was approximately 18–20 minutes. In both intervention groups, FR tempo was standardized using an audible metronome set at 40 BPM, and participants were instructed to synchronize their rolling rhythm with the metronome throughout the application. The investigator supervised the applications and provided verbal reminders when needed to maintain the target tempo and correct application technique. Pressure intensity was provided by the participant’s own body weight and was controlled subjectively using a target Numerical Rating Scale (NRS) score of 7/10, where 0 represented no pain or discomfort and 10 represented maximal pain or discomfort. At the beginning of the intervention, participants were instructed to perform the foam rolling application at a perceived pressure/pain intensity of 7/10. During the intervention, the investigator provided verbal reminders to maintain the target intensity. Participants regulated the applied pressure by shifting their body weight and modifying support from the upper limbs or non-treated limb according to the treated region. If a participant reported an intensity below or above the target, the participant was instructed to adjust the applied pressure until a perceived intensity of 7/10 was restored.

The control group performed seated passive rest for a duration equivalent to the total application time of the FR-B group. During this period, distracting elements such as phones and tablets were avoided.

#### Visual reaction time measurement.

Visual reaction time was assessed using the computer-based Human Benchmark Reaction Time Test. The test measures simple visual reaction in response to a change in the visual stimulus presented on the screen. During the test, participants were instructed to press the mouse button as quickly as possible when the red stimulus on the screen turned green. After each response, reaction time was automatically recorded in milliseconds, with lower values indicating faster reaction time. Because responses in the Human Benchmark Reaction Time Test are given by mouse click, only right-hand dominant participants were included in the study to reduce potential measurement differences related to hand dominance and mouse-use habits. Left-hand dominant participants were excluded from the study. Participants’ hand dominance was assessed based on the hand they preferred for daily activities and computer mouse use. Measurements were performed in a quiet room, with external distracting stimuli minimized. Participants were given standardized instructions to focus only on the visual stimulus on the screen and to respond as quickly as possible when the stimulus changed. The test was administered on a computer through an internet browser. To standardize testing conditions, the same hardware was used for all participants. Visual stimuli were presented using an MSI MAG 275QF monitor with a 27-inch screen size, 2560 × 1440-pixel WQHD resolution, 180 Hz refresh rate, and 0.5 ms response time. Participant responses were recorded using a Logitech G402 Hyperion Fury wired mouse. On the experimental testing day, the specific task procedure was explained, and participants completed three supervised practice trials that were not recorded. Following these practice trials, five valid pre-test trials were obtained from each participant. A trial was considered valid when the participant responded after the visual stimulus changed and the test produced a numerical reaction-time value. Anticipatory clicks made before the stimulus changed, which generated a “Too soon!” warning rather than a numerical value, were not included and were repeated until five valid trials had been obtained. For each participant, the lowest value among the five valid pre-test trials was defined as the primary best-trial score. The arithmetic mean and median of the same five valid trials were also calculated as alternative scoring measures for the sensitivity analyses. After the pre-test measurements, participants performed the relevant intervention or control protocol according to their group allocation. In the FR-A group, FR was applied for 30 seconds per region, whereas in the FR-B group, FR was applied for 60 seconds per region. In the intervention groups, participants were given 60 seconds of rest after the final application region was completed, after which they returned to the computer and post-test measurements were initiated. The control group rested passively for a duration equivalent to the total application time of the FR-B 60 s protocol, after which the same measurement procedure was repeated. Therefore, the elapsed time between the pre-test and post-test assessments was matched between the FR-B and control groups but was shorter in the FR-A 30 s group. During the post-test, five valid trials were again obtained from each participant using the same procedure and validity criterion. Anticipatory clicks were not included and were repeated until five valid numerical reaction-time values had been recorded. The lowest value was defined as the post-test best-trial score, and the arithmetic mean and median of the five valid post-test trials were calculated separately for the corresponding scoring-method sensitivity analyses. The reliability and within-session variability of the dominant-hand visual reaction time assessment were examined using the five valid recorded trials separately for the pre-test and post-test measurements. Single-measure reliability was evaluated using a two-way mixed-effects intraclass correlation coefficient (ICC) model for absolute agreement. Single-measure ICC values were reported with 95% confidence intervals. The standard error of measurement (SEM) was calculated as SD × √(1 − ICC), and the minimal detectable change at the 95% confidence level (MDC95) was calculated as SEM × 1.96 × √2. Within-subject coefficient of variation (CV) was calculated for each participant as the standard deviation of the five valid trials divided by the mean of the five valid trials and multiplied by 100. To minimize measurement variability, all assessments were conducted under standardized environmental and hardware conditions. In addition, participants completed both the familiarization session two weeks before testing and the practice trials on the measurement day before the recorded trials.

### Statistical analysis

Statistical analyses were performed using IBM SPSS Statistics version 31.0 (IBM Corp., Armonk, NY, USA) and JASP version 0.98.1. Continuous variables were summarized as mean ± standard deviation. For each participant, the lowest reaction time recorded across the five valid trials was defined as the best-trial score and retained as the primary scoring approach. To evaluate the sensitivity of the findings to the scoring method, the arithmetic mean and median of the five valid trials were also calculated separately for the pre-test and post-test measurements. Pre-test, post-test, and change values were reported descriptively for each group. Change was calculated as post-test minus pre-test; negative values therefore indicated lower post-test reaction time, whereas positive values indicated higher post-test reaction time. Change scores were not used as the basis for primary inferential conclusions.

The primary estimand was the between-group difference in dominant-hand post-test best-trial reaction time adjusted for the corresponding pre-test value. This estimand was initially evaluated using a one-way analysis of covariance (ANCOVA), with post-test reaction time as the dependent variable, group as the fixed factor, and pre-test reaction time as the covariate. Following a significant omnibus group effect, adjusted marginal means were compared using Bonferroni correction. The same ANCOVA framework was applied to the mean-based and median-based scores as scoring-method sensitivity analyses.

Model assumptions were evaluated separately for each scoring method. Normality was assessed using Shapiro–Wilk tests and Q–Q plots of the outcome distributions and standardized model residuals. Homogeneity of variance was examined using Levene’s test. Linearity of the baseline–post-test relationship was assessed by adding a quadratic baseline term, and homogeneity of regression slopes was evaluated using the group × baseline interaction. Standardized residuals, leverage values, and Cook’s distances were examined to identify potentially influential observations. No observation was excluded solely on the basis of these diagnostic indices.

Because the standard best-trial ANCOVA showed evidence of nonhomogeneous regression slopes and a nonlinear baseline–post-test relationship, an extended ANCOVA was fitted as an assumption-aware robustness analysis. The pre-test best-trial score was centered at the overall sample mean and entered into the model together with its squared term, group × centered baseline interaction, and group × centered baseline-squared interaction. Because variance heterogeneity and high-leverage observations were identified in the extended model, inference was additionally evaluated using 5,000 bias-corrected and accelerated (BCa) bootstrap replicates. Bootstrapped marginal means and Bonferroni-adjusted pairwise comparisons were estimated over the common observed baseline distribution and were interpreted in conjunction with the baseline interaction terms rather than as constant differences across all baseline values.

As a complementary analysis of raw pre-to-post trajectories, separate 2 × 3 mixed-design ANOVAs were conducted for the best, mean, and median scores, with time (pre-test and post-test) as the within-subject factor and group as the between-subject factor. These analyses evaluated time × group interactions and were considered complementary because they addressed between-group differences in change rather than the primary baseline-adjusted post-test estimand. Where change-score variances were heterogeneous, Welch and Brown–Forsythe omnibus tests and Games–Howell pairwise comparisons were examined. Mixed-design ANOVA results were not used to replace the primary ANCOVA-based estimand.

Effect sizes were reported as partial eta squared (partial η²), and effect estimates were accompanied by 95% confidence intervals where applicable. Statistical significance was set at p < .05.

## Results

Descriptive pre-test, post-test, and change values for dominant-hand best-trial visual reaction time are presented in Table 1.

**Table 1:** Dominant-hand pre-test, post-test and change values for best-trial visual reaction time by group.

Groups	n	Pre-test	Post-test	Change	95% CI for change
FR-A 30 s	34	172.76 ± 18.41	166.21 ± 13.46	−6.56 ± 22.34	−14.35 to 1.24
FR-B 60 s	34	182.29 ± 16.82	191.47 ± 17.81	9.18 ± 18.90	2.58 to 15.77
Control group	34	186.76 ± 17.98	186.65 ± 14.19	−0.12 ± 10.66	−3.84 to 3.60

*Note: Values are presented as mean ± standard deviation unless otherwise stated. Reaction time is expressed in milliseconds. Best-trial scores represent the lowest reaction time recorded across the five valid trials. Change was calculated as post-test − pre-test. Negative change values indicate lower post-test reaction time, whereas positive change values indicate higher post-test reaction time. CI: confidence interval.*

**Fig 1:**
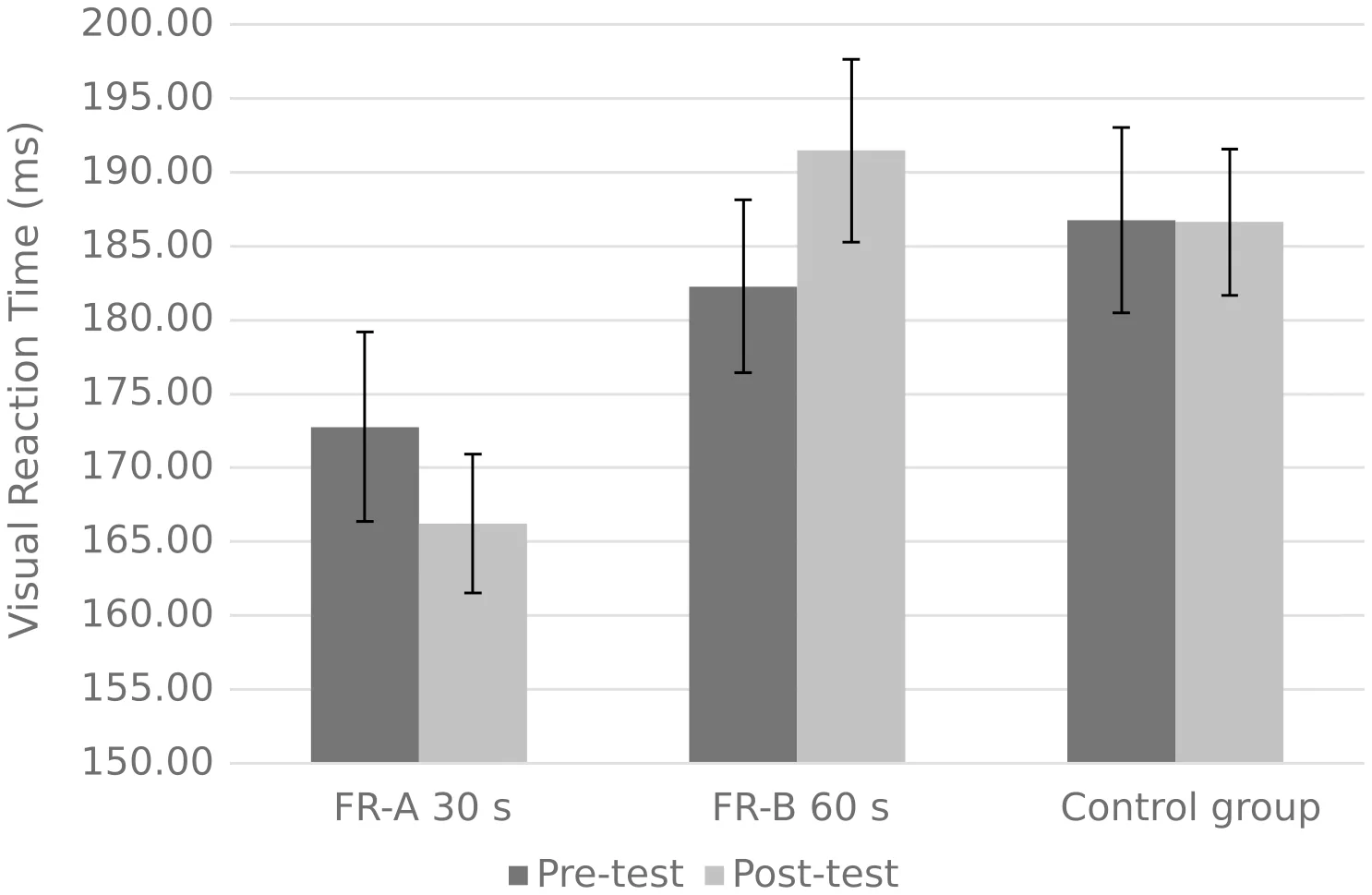
Mean pre-test and post-test best-trial visual reaction time values by group.

As shown in Table 1, the raw descriptive mean changes in best-trial reaction time were −6.56 ± 22.34 ms in the FR-A 30 s group, 9.18 ± 18.90 ms in the FR-B 60 s group, and −0.12 ± 10.66 ms in the control group. These change scores are presented descriptively with their corresponding variability and 95% confidence intervals and were not used as the basis for within-group inferential conclusions. The pre-test and post-test mean best-trial dominant-hand visual reaction time values by group are illustrated in Fig 1, with error bars representing 95% confidence intervals.

Single-measure ICC values across the five valid reaction time trials were 0.33 (95% CI: 0.24–0.43) for the pre-test measurements and 0.36 (95% CI: 0.27–0.46) for the post-test measurements, indicating poor single-measure reliability. The corresponding SEM values were 22.69 ms for the pre-test and 22.38 ms for the post-test, and the MDC95 values were 62.90 ms and 62.04 ms, respectively. The mean within-subject CV values were 9.86% and 9.60% for the pre-test and post-test measurements, respectively.

For the best-trial scoring approach, Shapiro–Wilk tests did not indicate significant departures from normality for the pre-test scores (W = .98, p = .26), post-test scores (W = .99, p = .92), or standardized model residuals (W = .99, p = .55). Visual inspection of the Q–Q plots was also considered acceptable. Levene’s test did not indicate significant heterogeneity of error variances, F(2, 99) = 2.54, p = .08. Standardized residuals ranged from −2.44 to 2.87, and no observation had an absolute standardized residual greater than 3 or a Cook’s distance greater than 1. Although some observations had relatively high leverage values, they were retained because no data-entry error or prespecified reason for exclusion was identified. However, the group × pre-test interaction was significant, F(2, 96) = 5.81, p < .01, partial η² = .11, indicating nonhomogeneous regression slopes. The quadratic pre-test term was also significant, F(1, 97) = 12.69, p < .01, indicating that the baseline–post-test relationship was not adequately represented by a strictly linear function. Accordingly, the standard ANCOVA results were interpreted cautiously and were followed by an extended ANCOVA that explicitly modeled these assumption violations.

In the initial standard ANCOVA, pre-test best-trial reaction time was significantly associated with post-test best-trial reaction time, F(1, 98) = 20.39, p < .01, partial η² = .17. After adjustment for the pre-test value, the omnibus group effect was statistically significant, F(2, 98) = 20.52, p < .01, partial η² = .30 (Table 2).

**Table 2:** Standard best-trial ANCOVA results for dominant-hand post-test reaction time.

Source	Type III SS	df	MS	F	p	Partial η²
Pre-Test Best-Trial Reaction Time	3977.83	1	3977.83	20.39	< .01	.17
Group	8005.33	2	4002.67	20.52	< .01	.30
Error	19115.97	98	195.06	–	–	–

*Note: The dependent variable was dominant-hand post-test best-trial visual reaction time. Dominant-hand pre-test best-trial visual reaction time was included in the model as a covariate. SS: sum of squares; MS: mean square; partial η²: partial eta squared.*

Following the significant omnibus group effect, Bonferroni-adjusted pairwise comparisons were performed using the adjusted marginal means. The adjusted means and pairwise comparisons are presented in Table 3.

**Table 3:** Adjusted means and Bonferroni-adjusted pairwise comparisons from the standard best-trial ANCOVA.

A. Adjusted Means				
Group	Adjusted mean	SE	95% CI	
FR-A 30 s	169.01	2.47	164.10 to 173.92	
FR-B 60 s	190.87	2.40	186.11 to 195.63	
Control group	184.45	2.44	179.60 to 189.30	
B. Bonferroni-Adjusted Pairwise Comparisons				
Comparison	Adjusted mean difference	SE	95% CI	p
FR-A – FR-B	−21.86	3.47	−30.31 to −13.41	< .01
FR-A – Control	−15.44	3.56	−24.12 to −6.76	< .01
FR-B – Control	6.42	3.41	−1.88 to 14.72	.19

*Note: Adjusted means were calculated after controlling for dominant-hand pre-test best-trial visual reaction time. Pairwise comparisons were based on adjusted means and adjusted using the Bonferroni correction. A negative mean difference indicates that the first group had a lower reaction time value than the comparison group. SE: standard error; CI: confidence interval.*

Under the standard ANCOVA model, the adjusted post-test mean was lower in FR-A than in both FR-B and the control group, whereas the difference between FR-B and the control group was not statistically significant (Table 3). However, because both the homogeneity-of-regression-slopes and linearity assumptions were violated, these adjusted differences should not be interpreted as constant across all baseline reaction-time values. The extended ANCOVA presented below was therefore used to examine the findings while accounting for baseline-dependent and nonlinear group differences.

To address the violations identified in the standard best-trial ANCOVA, an extended ANCOVA was fitted. The pre-test best-trial score was centered at the overall sample mean of 180.61 ms. Group, the centered pre-test score, its squared term, and the corresponding group interaction terms were included in the model. At the centered baseline value, the group term was statistically significant, F(2, 93) = 18.92, p < .01, partial η² = .29. The centered baseline term, F(1, 93) = 20.51, p < .01, partial η² = .18, and its squared term, F(1, 93) = 5.37, p = .02, partial η² = .06, were also significant. Importantly, both the group × centered baseline interaction, F(2, 93) = 3.35, p = .04, partial η² = .07, and the group × centered baseline-squared interaction, F(2, 93) = 3.48, p = .04, partial η² = .07, were statistically significant (Table 4). These interactions indicated that between-group differences varied as a function of baseline reaction time and could not be represented by a single constant group difference.

**Table 4:** Extended best-trial ANCOVA results for dominant-hand post-test reaction time.

Source	Type III SS	df	MS	F	p	Partial η²
Group	6185.00	2	3093.00	18.92	< .01	.29
Centered pre-test best-trial score	3354.00	1	3354.00	20.51	< .01	.18
Centered pre-test best-trial score²	878.20	1	878.20	5.37	.02	.06
Group × centered pre-test best-trial score	1096.00	2	548.20	3.35	.04	.07
Group × centered pre-test best-trial score²	1136.00	2	568.20	3.48	.04	.07
Residual	15200.00	93	163.50	—	—	—

*Note: The pre-test best-trial score was centered at the overall sample mean of 180.61 ms. Consequently, the group term represents between-group differences at this centered baseline value. Because the baseline interaction terms were significant, the group effect should not be interpreted as a constant difference across all baseline values. SS: sum of squares; MS: mean square; partial η²: partial eta squared.*

The standardized residuals from the extended model did not show a statistically significant departure from normality, W = .98, p = .08, and visual inspection of the residual Q–Q plot did not indicate a substantial deviation from normality. However, Levene’s test was statistically significant, F(2, 99) = 4.87, p = .01, indicating heterogeneity of error variances. Standardized residuals ranged from −2.45 to 2.18, with no absolute value greater than 3, and all Cook’s distances were below 1. Some observations at the extremes of the baseline distribution had relatively high leverage values, but none were excluded because no data-entry error or prespecified reason for exclusion was identified. Given the variance heterogeneity and high-leverage observations, the extended-model inference was additionally evaluated using 5,000 BCa bootstrap replicates.

The bootstrap marginal means, estimated over the common observed baseline distribution, were 167.60 ms for FR-A, 192.00 ms for FR-B, and 182.40 ms for the control group. Bonferroni-adjusted bootstrap comparisons indicated lower model-estimated reaction time in FR-A than in FR-B and the control group. The model-estimated reaction time in FR-B was higher than in the control group (Table 5).

**Table 5:** BCa bootstrap marginal means and pairwise comparisons from the extended best-trial ANCOVA.

A. Bootstrap marginal means				
Group	Marginal mean	SE	95% BCa CI	
FR-A 30 s	167.60	2.64	161.50 to 171.70	
FR-B 60 s	192.00	3.29	185.20 to 198.30	
Control group	182.40	1.84	178.80 to 186.00	
B. Bonferroni-adjusted bootstrap pairwise comparisons				
Comparison	Mean difference	SE	95% BCa CI	Adjusted p
FR-A − FR-B	−24.26	4.21	−33.29 to −17.21	< .01
FR-A − Control	−14.79	3.18	−22.01 to −9.59	< .01
FR-B − Control	9.52	3.60	2.63 to 16.99	.01

*Note: Estimates were based on 5,000 successful bootstrap replicates. Confidence intervals were calculated using the bias-corrected and accelerated method. Marginal means were estimated over the common observed baseline distribution. Pairwise p values were adjusted using the Bonferroni correction. Because the extended model included significant baseline interaction terms, these comparisons represent average model-based contrasts over the observed baseline distribution rather than constant differences at every baseline value. SE: standard error; BCa CI: bias-corrected and accelerated confidence interval.*

Thus, the between-group pattern observed with best-trial scoring remained evident after modeling the nonlinear and group-dependent baseline relationships and applying bootstrap inference. Nevertheless, the significant baseline interaction terms indicated that the magnitude and direction of the group differences were conditional on baseline reaction time. The consistency of these findings across alternative reaction-time scoring approaches was therefore subsequently examined using mean-based and median-based ANCOVAs.

As scoring-method sensitivity analyses, reaction time was additionally calculated using the arithmetic mean and median of the five valid trials. The descriptive values obtained using these alternative scoring approaches are presented in Table 6.

**Table 6:** Descriptive pre-test, post-test and change values for mean-based and median-based reaction time scores.

Scoring method	Group	Pre-test	Post-test	Change	95% CI for change
Mean	FR-A 30 s	197.37 ± 15.37	195.12 ± 14.43	−2.25 ± 17.13	−8.23 to 3.72
Mean	FR-B 60 s	207.04 ± 17.17	212.88 ± 20.90	5.84 ± 17.98	−0.43 to 12.12
Mean	Control group	210.58 ± 21.35	208.70 ± 18.61	−1.88 ± 16.09	−7.49 to 3.74
Median	FR-A 30 s	195.71 ± 16.76	194.71 ± 14.63	−1.00 ± 18.75	−7.54 to 5.54
Median	FR-B 60 s	206.62 ± 17.83	211.44 ± 22.51	4.82 ± 19.72	−2.06 to 11.70
Median	Control group	208.29 ± 21.70	207.76 ± 19.80	−0.53 ± 17.45	−6.62 to 5.56

*Note: Values are presented as mean ± standard deviation unless otherwise stated. Reaction time is expressed in milliseconds. Mean-based scores represent the arithmetic mean of the five valid trials, whereas median-based scores represent their median. Change was calculated as post-test − pre-test. Negative values indicate lower post-test reaction time, whereas positive values indicate higher post-test reaction time. CI: confidence interval.*

For the mean-based scores, Shapiro–Wilk tests indicated departures from normality for the pre-test scores (W = .97, p = .02), post-test scores (W = .97, p = .02), and standardized model residuals (W = .97, p = .01). The residual Q–Q plot indicated that the deviation occurred primarily in the upper tail. Levene’s test was not statistically significant, F(2, 99) = 0.14, p = .87. Neither the group × pre-test interaction, F(2, 96) = 1.40, p = .25, partial η² = .03, nor the quadratic pre-test term, p = .06, was statistically significant. Standardized residuals ranged from −2.14 to 3.73, with one observation exceeding an absolute value of 3. Although several observations exceeded the conservative Cook’s distance criterion of 4/n, all Cook’s distances were below 1 and no observation was excluded.

For the median-based scores, Shapiro–Wilk tests also indicated departures from normality for the pre-test scores (W = .97, p < .01), post-test scores (W = .97, p = .02), and standardized model residuals (W = .97, p = .03). The Q–Q plots indicated limited upper-tail deviations. Levene’s test was not statistically significant, F(2, 99) = 0.42, p = .66. The group × pre-test interaction, F(2, 96) = 1.89, p = .16, partial η² = .04, and the quadratic pre-test term, p = .14, were not statistically significant. Standardized residuals ranged from −2.56 to 2.72, and all Cook’s distances were below 1. No observation was excluded. Given the equal group sizes and nonsignificant Levene tests, the mean-based and median-based ANCOVA results were retained but interpreted in light of the residual normality findings.

In the mean-based ANCOVA, the corresponding pre-test score was a significant covariate, F(1, 98) = 44.17, p < .01, partial η² = .31. The adjusted group effect was also statistically significant, F(2, 98) = 5.39, p < .01, partial η² = .10. In the median-based ANCOVA, the pre-test score was a significant covariate, F(1, 98) = 36.55, p < .01, partial η² = .27, and the adjusted group effect was statistically significant, F(2, 98) = 3.53, p = .03, partial η² = .07 (Table 7).

**Table 7:** Mean-based and median-based ANCOVA sensitivity analyses.

A. ANCOVA Results							
Scoring method	Source	Type III SS	df	MS	F	p	Partial η²
Mean	Pre-test mean score	10166.00	1	10166.00	44.17	< .01	.31
Mean	Group	2480.95	2	1240.47	5.39	< .01	.10
Mean	Error	22554.83	98	230.15	—	—	—
Median	Pre-test median score	9973.07	1	9973.07	36.55	< .01	.27
Median	Group	1928.70	2	964.35	3.53	.03	.07
Median	Error	26738.49	98	272.84	—	—	—
B. Adjusted marginal means							
Scoring method	Group	Adjusted mean	SE	95% CI			
Mean	FR-A 30 s	199.40	2.68	194.10 to 204.70			
Mean	FR-B 60 s	211.70	2.61	206.60 to 216.90			
Mean	Control group	205.60	2.64	200.30 to 210.80			
Median	FR-A 30 s	198.90	2.92	193.10 to 204.70			
Median	FR-B 60 s	209.80	2.85	204.20 to 215.50			
Median	Control group	205.20	2.86	199.60 to 210.90			
C. Bonferroni-adjusted pairwise comparisons							
Scoring method	Comparison	Mean difference	SE	95% CI	Adjusted p		
Mean	FR-A − FR-B	−12.36	3.77	−21.54 to −3.18	< .01		
Mean	FR-A − Control	−6.21	3.84	−15.57 to 3.16	.33		
Mean	FR-B − Control	6.16	3.69	−2.84 to 15.15	.30		
Median	FR-A − FR-B	−10.94	4.12	−20.97 to −0.90	.03		
Median	FR-A − Control	−6.37	4.16	−16.49 to 3.76	.39		
Median	FR-B − Control	4.57	4.01	−5.20 to 14.33	.77		

*Note: Post-test reaction time was the dependent variable, group was the fixed factor, and the corresponding pre-test score was included as the covariate. Pairwise comparisons were based on adjusted marginal means and corrected using the Bonferroni method. A negative difference indicates that the first group had a lower adjusted reaction time than the comparison group. SS: sum of squares; MS: mean square; SE: standard error; CI: confidence interval; partial η²: partial eta squared.*

After Bonferroni correction, FR-A had a lower adjusted reaction time than FR-B under both mean-based scoring (mean difference = −12.36 ms, adjusted p < .01) and median-based scoring (mean difference = −10.94 ms, adjusted p = .03). Neither FR-A versus control nor FR-B versus control was statistically significant under either alternative scoring approach. Therefore, the FR-A–FR-B difference was consistent across the best-trial, mean, and median scoring methods, whereas the FR-A–control difference observed with best-trial scoring was not reproduced in the mean-based or median-based sensitivity analyses. This indicated that the comparison between FR-A and the control group was sensitive to the reaction-time scoring method.

As complementary analyses of the raw pre-to-post trajectories, separate 2 × 3 mixed-design ANOVAs were conducted for the best-trial, mean-based and median-based scores. The principal effect of interest was the time × group interaction. Because only two time points were included, the sphericity assumption was inherently satisfied.

For best-trial scoring, the main effect of time was not statistically significant, F(1, 99) = 0.22, p = .64, partial η² = .00. However, the time × group interaction was statistically significant, F(2, 99) = 6.58, p < .01, partial η² = .12, indicating different pre-to-post trajectories among the groups. The between-subject group effect was also statistically significant, F(2, 99) = 17.56, p < .01, partial η² = .26. In contrast, the time × group interaction was not statistically significant for either mean-based scoring, F(2, 99) = 2.43, p = .09, partial η² = .05, or median-based scoring, F(2, 99) = 1.02, p = .36, partial η² = .02 (Table 8).

**Table 8:** Complementary mixed-design ANOVA results by reaction-time scoring method.

Scoring method	Effect	F	df	p	Partial η²
Best trial	Time	0.22	1, 99	.64	.00
Best trial	Time × group	6.58	2, 99	< .01	.12
Best trial	Group	17.56	2, 99	< .01	.26
Mean	Time	0.11	1, 99	.74	.00
Mean	Time × group	2.43	2, 99	.09	.05
Mean	Group	8.11	2, 99	< .01	.14
Median	Time	0.35	1, 99	.55	.00
Median	Time × group	1.02	2, 99	.36	.02
Median	Group	7.30	2, 99	< .01	.13

*Note: Time comprised the pre-test and post-test measurements, and group comprised FR-A 30 s, FR-B 60 s, and the control group. The time × group interaction evaluated whether the pre-to-post trajectory differed among the groups. Partial η²: partial eta squared.*

In the best-trial analysis, the within-group pre-to-post effect was statistically significant in FR-B, F(1, 33) = 8.02, p < .01, but not in FR-A, F(1, 33) = 2.93, p = .10, or the control group, F(1, 33) = 0.00, p = .95. The corresponding raw mean changes were 9.18 ms in FR-B, −6.56 ms in FR-A, and −0.12 ms in the control group. However, Levene’s test indicated heterogeneous change-score variances, F(2, 99) = 3.68, p = .03. The overall between-group difference in change was therefore additionally examined using robust tests and remained statistically significant with both the Brown–Forsythe test, F(2, 79.69) = 6.58, p < .01, and Welch’s test, F(2, 59.36) = 5.25, p < .01.

Games–Howell comparisons indicated that the best-trial change in FR-B differed from both FR-A (FR-A − FR-B = −15.74 ms, 95% CI: −27.77 to −3.70, p < .01) and the control group (control − FR-B = −9.29 ms, 95% CI: −18.27 to −0.32, p = .04). The change in FR-A did not differ significantly from the control group (control − FR-A = 6.44 ms, 95% CI: −3.83 to 16.71, p = .29). Change-score variances were homogeneous for the mean-based, F(2, 99) = 0.05, p = .95, and median-based scores, F(2, 99) = 0.11, p = .90; therefore, the nonsignificant time × group interactions for these scoring approaches could not be attributed to heterogeneous change-score variances.

Overall, a statistically significant difference in pre-to-post trajectories was observed only under best-trial scoring and was primarily characterized by higher post-test reaction time in FR-B relative to its pre-test value and by the separation of FR-B from FR-A and the control group. This interaction was not reproduced using mean-based or median-based scoring. Accordingly, the complementary mixed-design ANOVAs further indicated that the pre-to-post trajectory findings were sensitive to the reaction-time scoring method and were interpreted separately from the baseline-adjusted ANCOVA estimand.

## Discussion

To the best of our knowledge, no previous study has examined the acute effects of duration-specific, self-administered foam rolling on visual reaction time in youth athletes under field-based conditions. The present findings suggest that acute reaction-time responses to self-administered FR may vary according to application duration, baseline reaction time, and the method used to summarize repeated reaction-time trials. In the standard best-trial ANCOVA, FR-A showed lower adjusted post-test reaction time than both FR-B and the control group; however, the homogeneity-of-regression-slopes and linearity assumptions were violated. The extended best-trial model confirmed that between-group differences varied according to baseline reaction time, and its bootstrap comparisons supported separation among the three groups over the common observed baseline distribution. In the mean-based and median-based sensitivity analyses, only the difference between FR-A and FR-B remained statistically significant, whereas neither intervention group differed significantly from the control group. Similarly, a significant time × group interaction was observed only under best-trial scoring and was primarily characterized by higher post-test reaction time in FR-B relative to its pre-test value. Therefore, the most consistent finding across scoring approaches was the difference between the 30-second and 60-second FR protocols, whereas the comparison between FR-A and the control group was sensitive to the scoring method. These findings support a duration-dependent response but do not establish a scoring-independent benefit of the 30-second protocol over the control condition.

The positive effects of FR on joint range of motion, muscle stiffness, and flexibility have been widely reported in the literature [28–30]. However, evidence regarding multifactorial performance outcomes such as reaction time remains limited, and the potential contributions of central and peripheral mechanisms should be interpreted cautiously. Sawamura and Mikami examined the acute effects of a fascial stimulation technique applied by a healthcare professional on reaction time and neuromuscular performance in healthy adults in 2020 [23]. In that study, reaction time decreased from 253.9 ± 31.4 ms to 228.6 ± 31.2 ms after the application; motor time decreased from 88.8 ± 21.8 ms to 62.1 ± 25.8 ms; time to peak activity decreased from 81.9 ± 20.6 ms to 63.5 ± 24.8 ms; and time to peak force decreased from 172.6 ± 24.0 ms to 143.4 ± 26.2 ms. Following the 210-second fascial manipulation intervention, significant improvements compared with baseline values were reported in parameters such as reaction time, motor time, time to peak activity, and time to peak force, both acutely and one week after the intervention. Moreover, no statistically significant difference was found between the acute post-application measurements and the measurements taken one week later, indicating that the initial improvements were maintained for one week. In contrast, the absence of a change in premotor time suggests that the intervention may have acted through peripheral mechanisms rather than central processing mechanisms.

In the present study, the lower post-test best-trial reaction time observed descriptively in FR-A was directionally consistent with the findings of Sawamura and Mikami [23]. However, this pattern should not be interpreted as a confirmed within-group improvement or a scoring-independent benefit over the control condition, because the FR-A–control difference was not reproduced using mean-based or median-based scoring and the best-trial mixed-design analysis did not show a statistically significant within-group pre-to-post effect in FR-A. Therefore, the present findings provide only partial support for consistency with the earlier study. If a short-duration response exists, it could potentially be related to changes in the mechanical properties of the muscle–fascia unit or peripheral neuromuscular responses; however, these mechanisms were not directly measured in the present study.

One literature-based hypothesis for such a response is that pressure applied during FR may activate sensory receptors in the skin and muscle. It has been suggested that such mechanical stimulation may facilitate muscle relaxation by inhibiting sympathetic nervous system activity and may support neuromuscular response organization by increasing afferent sensory input [20,28]. On the other hand, the fact that the intervention in the aforementioned study was manually applied by a therapist and involved upper-extremity manipulation differs methodologically from the present study, which included self-administered fascial stimulation through FR and whole-body applications. This may be considered a factor that could explain differences in observed performance responses when the duration and method of application vary. In another study examining the relationship between fascial stimulation and reaction time, Kuruma et al. compared the acute effects of MFR and static stretching techniques on joint range of motion, muscle stiffness, and reaction time in healthy individuals in 2013 [22]. They reported that, after MFR protocols applied to the m. quadriceps femoris and hamstring muscles for 8 minutes, premotor time decreased from 267.4 ± 66.5 ms to 223.3 ± 46.7 ms following quadriceps application and from 267.2 ± 86.5 ms to 200.7 ± 45.3 ms following hamstring application. Similarly, total reaction time decreased from 443.9 ± 82.9 ms to 383.6 ± 66.0 ms following quadriceps application and from 412.3 ± 65.5 ms to 342.4 ± 48.7 ms following hamstring application, and these changes were also reported to be significant compared with the control group. In contrast, although total reaction time decreased from 388.8 ± 76.9 ms to 368.4 ± 56.3 ms in the static stretching group, this change was not statistically significant. These findings indicate that MFR applications may provide not only mechanical benefits but also positive effects on the speed of neuromuscular responses. In the present study, raw descriptive values showed that visual reaction time in the FR-A group, which performed the 30 s per region FR protocol, decreased from 172.76 ± 18.41 ms to 166.21 ± 13.46 ms, corresponding to a descriptive change of −6.56 ms. This change should not be interpreted as a within-group statistically significant improvement, because the primary inferential analysis was based on baseline-adjusted between-group comparisons. This descriptive pattern is partly consistent with the reaction time improvements reported by Kuruma et al. in the MFR groups. The fact that the MFR application duration in the study by Kuruma et al. was limited to 8 minutes represents an important methodological difference that may help explain the opposite-direction responses observed in the present study following SMR protocols applied for different durations. This suggests that the duration and mode of fascial stimulation may be critical factors determining acute neuromuscular performance responses. On the other hand, the MFR protocols used in the studies by Kuruma et al. (2013) and Sawamura and Mikami (2020) involved therapist-assisted manual interventions applied to isolated regions, whereas the FR protocol used in the present study was based on self-administered FR, an SMR approach commonly used by athletes in pre-competition and pre-training warm-up routines, and involved a whole-body protocol [22,23]. In this respect, the present study examined the effects of a warm-up strategy with high field applicability on acute neuromotor outcomes, rather than those of a therapist-assisted manual intervention. Although the present study focused on the pre-exercise use of FR, this intervention is also used as a recovery modality. In recovery settings, predictive models integrating athlete characteristics, training load, recovery status, and injury history may help individualize FR protocols within broader injury-prevention strategies [31]. The FR protocol used in the present study was implemented under standardized laboratory conditions. Application duration per region was controlled according to group allocation, target tempo was guided using a 40 BPM metronome, and perceived pressure intensity was standardized using a target NRS score of 7/10. These procedures were used to support protocol consistency across participants while avoiding direct assumptions about objectively measured application variability.

In this context, the separation observed between the 30-second and 60-second protocols suggests that application duration may be relevant to acute neuromotor responses, although the strength of this pattern depended on the reaction-time scoring method. From a broader analytical biomechanics perspective, recent modeling work has illustrated that the interpretation of mechanically induced outcomes depends on clearly defined assumptions regarding body-segment geometry, movement trajectories, and mechanical constraints [32]. Accordingly, the present findings are interpreted as duration-related reaction-time responses rather than as direct evidence of specific mechanical or neuromuscular mechanisms. Supporting the broader importance of stimulation duration, Çalışkan et al. (2019) examined the effects of different durations of static stretching applied to the rectus femoris muscle on muscle stiffness in adolescent athletes and reported that 2 minutes of stretching did not produce a marked reduction in muscle stiffness, whereas 5 minutes of stretching significantly reduced muscle stiffness compared with resting conditions. This finding indicates that the duration of mechanical stimulation may influence the mechanical response of muscle–tendon and fascial tissues [33]. In the present study, raw best-trial reaction time in FR-B increased from 182.29 ± 16.82 ms to 191.47 ± 17.81 ms, corresponding to a descriptive change of 9.18 ms. The complementary best-trial mixed-design analysis identified a significant within-group pre-to-post effect in FR-B and a separation of FR-B from FR-A and the control group in the Games–Howell comparisons. However, the time × group interaction was not statistically significant using mean-based or median-based scoring. Moreover, although the standard best-trial ANCOVA did not identify a significant FR-B–control difference, the extended bootstrap comparison over the common baseline distribution did; this contrast remained conditional on baseline reaction time and was not reproduced in the alternative scoring analyses. Therefore, the findings do not establish a general scoring-independent performance decrement following the 60-second protocol, but they raise the possibility that longer-duration FR may be associated with a less favorable acute reaction-time response under some analytical and baseline conditions.

Previous studies have reported that prolonged FR applications may be associated with temporary reductions in performance [34–36]. Although the present study did not directly measure neurophysiological mechanisms, previous literature has discussed mechanisms such as H-reflex suppression, reduced motor neuron excitability, and increased parasympathetic activity as possible explanations for acute reductions in performance [37–39]. Therefore, these mechanisms are considered only as a literature-based explanatory framework for interpreting the observed response pattern. In the present study, only the dominant extremity was evaluated. The dominant extremity may receive greater neural input within the motor system, which may have allowed the acute responses to FR applications to be observed more clearly in this extremity [40].

The raw descriptive values in the control group showed minimal pre-test to post-test change. The standard and extended best-trial analyses indicated lower model-estimated post-test reaction time in FR-A than in the control group. However, this difference was not reproduced using mean-based or median-based ANCOVA, and the best-trial mixed-design analysis did not identify a significant difference in change between FR-A and the control group. Consequently, the evidence for a more favorable response to the 30-second protocol relative to the control condition was dependent on the scoring method and analytical framework and should not be interpreted as a robust protocol benefit. This comparison is further limited by the fact that the elapsed time between pre-test and post-test was not fully matched between FR-A and the control condition and that the study did not include a sham or attention control condition. Consequently, the present findings do not permit a definitive conclusion that the 30-second FR protocol is superior to passive rest, because the observed difference cannot be separated fully from the potential influence of the unequal elapsed time between assessments.

Previous literature has proposed that short-duration FR may alter afferent sensory input through peripheral tissue stimulation, whereas longer-duration applications may be associated with neural inhibition or fatigue-like responses [4,7,8,41–44]. However, these mechanisms were not directly assessed in the present study and should be regarded as possible literature-based explanations rather than confirmed mechanisms underlying the observed findings.

### Limitations

Several limitations should be considered when interpreting the findings. First, the study was conducted only in right-hand-dominant male U16–U18 youth basketball players; therefore, the generalizability of the results to female athletes, left-hand-dominant athletes, different age groups, different sports, or different performance levels is limited. Recent research in elite U-18 female basketball players has demonstrated that structured interventions can produce neuromuscular adaptations in physical performance outcomes [45]. However, such findings do not establish whether comparable responses occur in perceptual–motor outcomes such as reaction time. Therefore, whether the duration-related acute reaction-time responses observed in the present study also apply to female youth basketball players remains an open question requiring direct investigation. Second, only single-session acute responses were evaluated in this study; therefore, no inference can be made regarding the chronic effects of FR applications or adaptations related to repeated applications. Third, the physiological mechanisms that could explain changes in reaction time were not directly measured. The absence of assessments such as muscle stiffness, blood flow, muscle oxygenation, EMG activity, or reflex responses limits interpretations regarding the central and peripheral effects of FR application.

The elapsed time between the pre-test and post-test assessments was matched between FR-B and the control group but was shorter in FR-A, and exact participant-level assessment intervals were not recorded. Therefore, differences attributed to FR duration cannot be completely separated from differences in the elapsed time before post-test assessment, including possible differences in recovery duration, repeated-test learning, or arousal decay. In addition, the absence of a sham or attention control condition limits the ability to distinguish intervention-specific effects from expectancy, attention, or other nonspecific effects. The computer-based task measured simple visual reaction time under standardized conditions and did not include stimulus discrimination, response selection, decision-making, or sport-specific perceptual demands. Consequently, the findings should not be generalized directly to basketball decision-making or complex on-court reaction performance.

The statistical findings were also sensitive to model assumptions and the method used to summarize the five reaction-time trials. The standard best-trial ANCOVA violated the homogeneity-of-regression-slopes and linearity assumptions, and the extended model indicated that between-group differences varied according to baseline reaction time. Because variance heterogeneity and high-leverage observations were identified, BCa bootstrap inference was used as an additional robustness evaluation. However, bootstrap inference does not remove the influence of these observations or eliminate the conditional nature of the model estimates. The mean-based and median-based ANCOVAs met the homogeneity-of-regression-slopes, linearity, and variance-homogeneity assumptions but showed departures from residual normality. Most importantly, the FR-A–control difference observed with best-trial scoring was not reproduced using mean-based or median-based scoring, and the significant time × group interaction was also limited to the best-trial analysis. Therefore, the comparison between FR-A and the control group should be considered scoring-sensitive, whereas the FR-A–FR-B adjusted difference was the most consistent finding across the three scoring approaches.

Although the visual reaction time assessment was standardized and based on five valid recorded trials, the single-measure ICC values indicated limited reliability and trial-to-trial variability. This finding should be considered in the context of the sensitivity of millisecond-level reaction time measurements to normal within-participant fluctuations and the relatively limited between-participant variability expected in a homogeneous youth athlete sample. The SEM values were 22.69 ms at pre-test and 22.38 ms at post-test, while the corresponding MDC95 values were 62.90 ms and 62.04 ms, respectively. These measurement-error estimates indicate that small pre-test-to-post-test changes should not be interpreted as precise individual-level changes. However, they do not directly invalidate the group-level statistical comparisons, which were based on the full sample and were evaluated using multiple scoring approaches and additional robustness analyses. Nevertheless, the practical meaning of small millisecond-level effects should be interpreted cautiously, particularly when findings were not consistent across the best-trial, mean-based, and median-based scoring methods.

Although pressure intensity was standardized using a target NRS score of 7/10, achieved NRS values were not recorded at the participant or group level. Therefore, potential variability in applied pressure between participants or between intervention groups could not be quantified. Participant-level adherence, rolling amplitude, and achieved tempo were not quantitatively recorded; therefore, variability in protocol execution beyond investigator supervision could not be evaluated.

## Conclusion

In conclusion, acute dominant-hand visual reaction-time responses to self-administered FR differed between the 30-second and 60-second protocols, but the observed pattern was conditional on baseline reaction time and the scoring method used to summarize repeated trials. The most consistent finding was a lower adjusted post-test reaction time in FR-A than in FR-B across the best-trial, mean-based, and median-based ANCOVA analyses. In contrast, the FR-A–control difference was observed only with best-trial scoring and was not reproduced in the mean-based or median-based sensitivity analyses. Similarly, a significant difference in pre-to-post trajectories was identified only in the best-trial mixed-design analysis and was primarily characterized by higher post-test reaction time in FR-B. Therefore, the findings do not establish a scoring-independent benefit of the 30-second protocol over the control condition or a general detrimental effect of the 60-second protocol. These results should be considered preliminary and are insufficient to support a firm recommendation for pre-training or pre-competition practice. Further studies using fully time-matched assessments, sham or attention control conditions, more reliable and sport-specific reaction-time measures, diverse athlete samples, and direct neuromuscular or physiological measurements are needed to clarify the practical and mechanistic significance of duration-dependent FR responses.

## Supporting information

S1 Data Anonymized dataset underlying the study findings. (XLSX)
